# 743 Educate Before You Operate: Improving the Informed Consent Process

**DOI:** 10.1093/jbcr/irad045.218

**Published:** 2023-05-15

**Authors:** Brian Piatkowski, Jeanne Lee, Eli Strait

**Affiliations:** UC San Diego Regional Burn Center, San Diego, California; UC San Diego Health, San Diego, California; UC San Diego Health, San Diego, California; UC San Diego Regional Burn Center, San Diego, California; UC San Diego Health, San Diego, California; UC San Diego Health, San Diego, California; UC San Diego Regional Burn Center, San Diego, California; UC San Diego Health, San Diego, California; UC San Diego Health, San Diego, California

## Abstract

**Introduction:**

The informed consent process is a daily task for providers with a surgical patient population. In the burn population, informed consent is often presented by an intern or resident physician. Consent is comprised of surgical debridement with a multitude of options for coverage of their wounds. The current state of practice is a verbal overview of all the possible procedures that may be done to the patient’s wound(s). Patients often verbalize feeling overwhelmed with the amount of information on the consent and often have questions related to their procedure just before the brief of the operative case.

**Methods:**

An educational video was developed that detailed the surgical procedure and the potential burn wound coverings. A 3-question survey was given to patients who have already been through the informed consent process. Survey metrics examined knowledge of consent when signed, the satisfaction of verbal explanation, and if a video would increase understanding. The video was given to the same patients to watch. After the video was viewed, those patients were then again surveyed. Providers were also given a 3-question survey before viewing the video. Survey metrics examined comfort of consent, knowledge of procedures, and if the video would increase patient understanding of consent topics. The providers were then surveyed after watching the video.

**Results:**

Initial post-implementation data shows that patients and providers have increased comfort and knowledge in the informed consent process. Patients show an 80% increase in understanding of consent, a 72% increase in satisfaction with video vs verbal overview, and a 97% increase in satisfaction with material viewed. Provider data shows a 65% increase in the comfort of consent, a 64% increase in knowledge of procedures, and a 97% increase that the video will help patients understand their consent. This shows that this evidence-based project is an improvement from the current standard of practice.

**Conclusions:**

The informed consent process is an opportunity for providers and patients to have a moment of discussion. It is a pivotal point in which a patient and a provider determine the next step of their care. The patient must have clear communication and education regarding the procedures to which they are consenting. It is also a discussion where a provider can provide education and support at what is an overwhelming time for patients in their hospital stay.

**Applicability of Research to Practice:**

Implementation of a standardized audio/video teaching method for burn surgical patients is an effective way to increase patient and provider satisfaction regarding the informed consent process. Implementing this educational tool is a cost-effective and simple way to educate burn patients before their surgical procedures. There is an overall improvement in patient satisfaction and increased satisfaction in the providers who obtain the consent.

